# History and prospects of Nagashima‐type palmoplantar keratosis, the most common palmoplantar keratoderma in east Asian populations

**DOI:** 10.1111/1346-8138.17552

**Published:** 2025-01-03

**Authors:** Akiharu Kubo

**Affiliations:** ^1^ Division of Dermatology, Department of Internal Related Kobe University Graduate School of Medicine Kobe Japan

**Keywords:** Mal de Meleda, Nagashima‐type palmoplantar keratosis, palmoplantar, transgrediens keratoderma

## Abstract

Nagashima‐type palmoplantar keratosis (NPPK) has been shown to represent a form of autosomal recessive palmoplantar keratosis due to biallelic pathological variants of *SERPINB7*, which encodes a serine protease inhibitor expressed in the epidermis. Approximately 10 years have elapsed since NPPK was demonstrated to be an independent genetic disease, and the most prevalent palmoplantar keratoderma (PPK) in East Asian countries due to a high prevalence of founder mutations in *SERPINB7*. Since then, it has become evident that biallelic pathological variants of *SERPINA12*, which encodes a serine protease inhibitor expressed in the epidermis, can also manifest symptoms analogous to those of NPPK. Furthermore, a pathological variant of *SERPINB7* was identified as a risk factor for the development of atopic dermatitis in a genome‐wide association study (GWAS) of atopic dermatitis, indicating that the frequent co‐occurrence of NPPK and atopic dermatitis is not a mere coincidence. Despite the documentation of NPPK cases in Japan since the 1970s, there have been no reports of individuals with similar symptoms from other regions, including Europe and the USA. Consequently, the existence and independence of the disease remained uncertain until its genetic cause was identified. The disease's independence was established through the accumulation of data on affected individuals, including the provision of accurate descriptions of their symptoms, which enabled the identification of the genetic cause. This review presents a comprehensive overview of the history and prospects of NPPK with a particular focus on the history of the process of establishing NPPK as an independent disease.

## 1 PALMOPLANTAR KERATODERMA WITH “TRANSGREDIENS”

There is a wide variety of diseases, ranging from hereditary to inflammatory, that manifest hyperkeratosis of the palms and soles. In the differential diagnosis based on clinical manifestations, the main factors to be considered are the degree and morphology of hyperkeratosis, the extent and distribution of hyperkeratosis, the presence or absence of associated symptoms, and family history of the disease. The presence or absence of cutaneous manifestations other than palmoplantar lesions, age at onset, the extent and degree of progressive hyperkeratosis, and histopathological diagnosis are also useful in the diagnosis.^1^, ^2^

**FIGURE 1:**
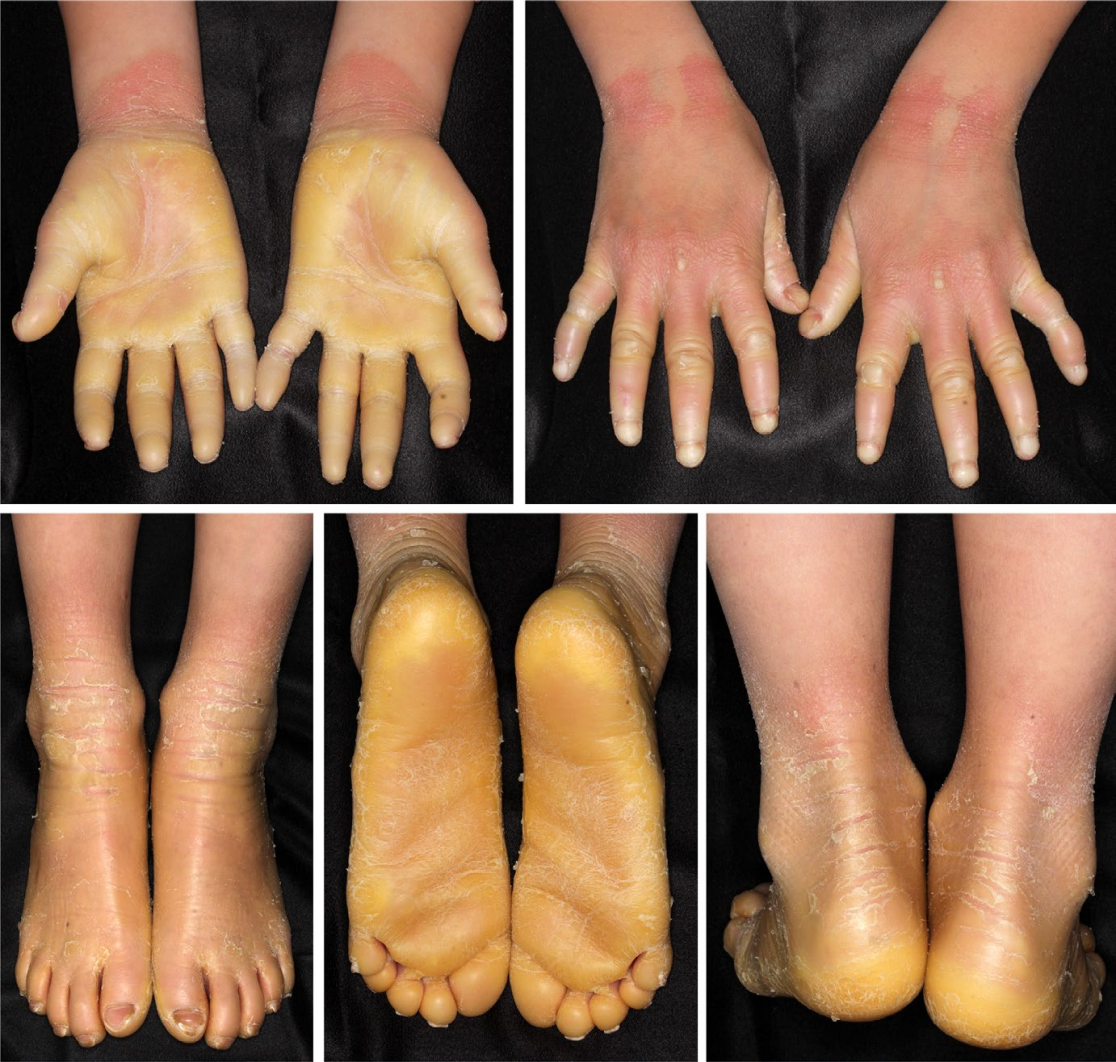
Severe palmoplantar hyperkeratosis with transgrediens in a case of Mal de Meleda. Eight‐year‐old female with a homozygous c.256G > A (p.Gly86Arg) variation in *SLURP1*.

The term “transgrediens” is employed to describe a congenital palmoplantar keratoderma (PPK) in which the hyperkeratotic symptom is not confined to the palms and soles but rather extends to the dorsal skin of fingers and toes, the dorsal surface of hands and feet, the inner wrist and Achilles tendon area, elbow, and knee. The most renowned PPK with transgrediens is Mal de Meleda (MIM#248300), which is caused by *SLURP1* pathogenic variants and now includes Gamborg‐Nielsen (Norrbotten) PPK. Other PPK with transgrediens include Bothnia‐type PPK (MIM 600231) caused by *AQP5* variants, KID syndrome (MIM 1482100) caused by *GJB2* variants, and Papillon‐Lefevre syndrome (MIM 245000) caused by *CTSC* variants. However, in the 1970s, the only representative PPK with transgrediens was Mal de Meleda. This disease is characterized by early childhood onset, severe and progressive keratinization, extension of the lesions from the wrists to the forearms and from the dorsal surfaces of the feet to the ankles, erythema of the elbows, knees, and mouth, and, occasionally, finger dissection (Figure 1). Mal de Meleda is caused by biallelic pathogenic variants of *SLURP1*.^3^ Several pathogenic variants are shared by Algerian and Croatian populations. The pathogenic variants carried by the common ancestors of these populations have been passed down and spread from generation to generation (founder effect), resulting in a high prevalence of affected individuals in rural areas, including the island of Mljet (Mereda Island) in Croatia.

## 2 INITIAL DESCRIPTION OF PPK COMMON IN JAPANESE, DIFFERENT FROM MAL DE MELEDA, BY MASAJI NAGASHIMA

**FIGURE 2:**
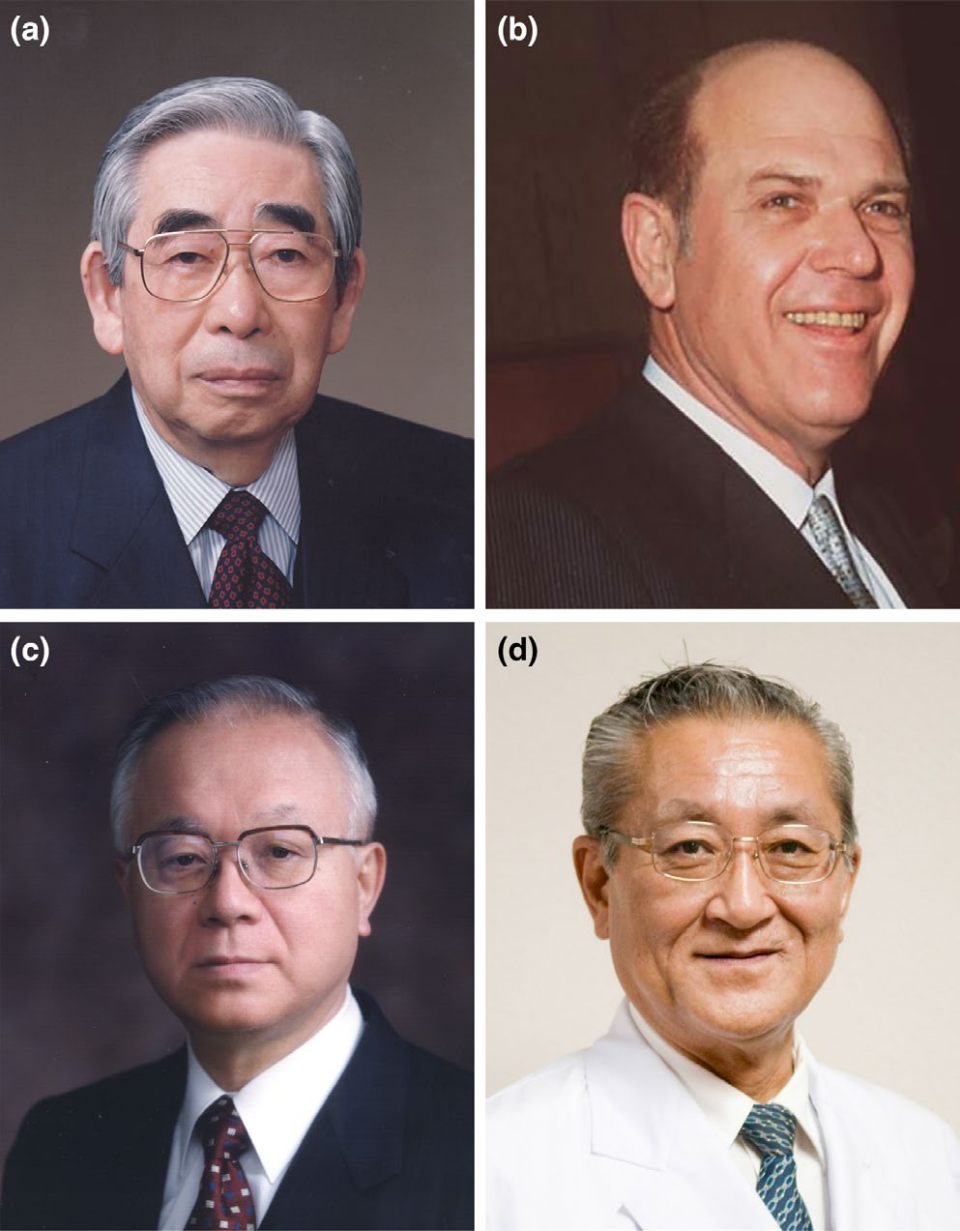
Key individuals in the establishment of Nagashima‐type palmoplantar keratosis. (a) Professor Masaji Nagashima (courtesy of the Department of Dermatology, Kyorin University Faculty of Medicine); (b) Professor Urs Schnyder (courtesy of the Department of Dermatology, University Hospital Heidelberg); (c) Professor Isao Hashimoto (courtesy of the Department of Dermatology, Hirosaki University Faculty of Medicine), and (d) Professor Yoshihiko Mitsuhashi (courtesy of the Department of Dermatology, Tokyo Medical University Hospital).

Although it is now known that Mal de Meleda is extremely rare in the Japanese population, this was not apparent in the 1970s. Moreover, clinical photographs and information about Mal de Meleda were not readily accessible in Japan at that time. Consequently, until the 1970s, isolated cases of PPK with transgrediens were diagnosed as Mal de Meleda in Japan. It was the late Masaji Nagashima (Figure 2a), Associate Professor of Dermatology at Keio University in Japan (subsequently Professor at Kyorin University in Japan), who first noticed that the symptoms of PPK commonly encountered in Japanese dermatological outpatient clinics differed from those documented in the medical literature for Mal de Meleda. Thus, he was the first to recognize the disease. In the section on PPK in the “Handbook of Clinical Genetics,” published in Japanese in 1977, Nagashima described the syndrome as follows: “A few cases [of PPK] have been reported in Japan as Mal de Meleda. In fact, PPK, which often occurs in families with evidence of consanguineous marriage, presents with erythematous hyperkeratosis and a distribution of lesions similar to that of Mal de Meleda. It is often complicated by hyperhidrosis of the palms and soles. However, many of these cases are milder and less progressive than Mal de Meleda, and, therefore, it is unlikely that these forms are consistent with Mal de Meleda. It is, therefore, proposed that these be designated as Meleda‐type PPK, which is distinct from Mal de Meleda”.^4^ This was the first delineation of the clinical manifestations of Nagashima‐type palmoplantar keratosis (NPPK). Although the description is only six lines in Japanese in the textbook, it is concise and precise, representing the essence of descriptive dermatology.

## 3 KERATOSIS PALMOPLANTARIS NAGASHIMA COINED BY MITSUHASHI AND HASHIMOTO

In 1982, Urs Walter Schnyder (Figure 2b), a Professor at Heidelberg University, West Germany who was a leading researcher into hereditary skin diseases at the time,^5^ was invited to Hirosaki University by Isao Hashimoto (Figure 2c), who had previously studied in Schnyder's department. Schnyder was engaged in research on Mal de Meleda on the island of Mljet and had a profound understanding of the disease.^6^ Hashimoto presented a case of Japanese palmoplantar keratosis to Schnyder, suggesting that it might be Mal de Meleda. However, Schnyder replied, “That is unlikely.” He stated emphatically, as recalled by Hashimoto:^7^ “This mild case of PPK is not Mal de Meleda,” and the individual was diagnosed with a form of “keratosis palmoplantaris transgrediens” but which was significantly less severe than Mal de Meleda. Although the case was an isolated one, given that the parents of the patient were cousins it was postulated that it was a recessive genetic disorder rather than a de novo‐appearing dominant genetic disorder. However, this case was not reported since the mode of inheritance could not be determined based on the available evidence. Subsequently, Hashimoto and his colleague Yoshihiko Mitsuhashi (Figure 2d) identified a second case, that of a brother and sister presenting with similar symptoms while their parents had no symptoms. This case was presented at the 71st Swiss Society for Dermatology and Venereology meeting as a novel form of PPK.^8^ The condition was designated “keratosis palmoplantaris Nagashima” (NPPK) and was described in detail in the Japanese Journal of Dermatological Practice.^9^ In their review of the literature, they identified the aforementioned description by Nagashima as a relevant reference. Hashimoto and Mitsuhashi considered the designation “Meleda‐type” coined by Nagashima to be potentially misleading. In light of these considerations, they coined the designation “Nagashima‐type,” thereby paying homage to Nagashima's invaluable insights. The details of the naming process are meticulously documented in the record of the discussion with Nagashima at a Japanese conference.^10^ Subsequent to this, Nagashima took the opportunity to reflect on those discussions and express his profound gratitude to Hashimoto and Mitsuhashi in a Japanese publication.^11^

## 4 PROPOSAL OF THE GENETIC INDEPENDENCE OF NPPK

Subsequently, this condition was documented in Japanese medical literatures as either a Meleda‐type or Nagashima‐type PPK. Meanwhile, as mentioned in the review article by Lucker et al., there have been no case reports of the disease from other countries.^12^ The paucity of case reports outside of Japan was thought to be due to the lack of English‐language sources providing detailed descriptions of symptoms. However, the discovery of pathogenic variations in *SERPINB7* that cause NPPK has revealed that there are almost no carriers of pathogenic variations in Caucasians, except in Finland.

To ascertain whether NPPK constitutes a discrete disease entity or a milder form of Mal de Meleda, it was essential to identify the pathogenic genetic variations for Mal de Meleda and NPPK. In 2001, three homozygous variations in *SLURP1* were identified in the individuals of Mal de Meleda, and it was confirmed that Mal de Meleda is an autosomal recessive genetic disorder caused by pathogenic variations in the gene encoding the secreted Ly6/uPAR‐related protein, SLURP1.^3^ It is, therefore, pertinent to enquire whether individuals with NPPK have variations in *SLURP1*. Yoshiki Tokura and Kenji Kabashima identified the absence of variations in the exon region of *SLURP1* in a single case of NPPK. They presented this case of NPPK accompanied by comprehensive clinical descriptions.^13^ Subsequently, multiple case reports by Tokura and Kabashima introduced hereditary PPK internationally as a distinct entity, namely NPPK, which is distinct from Mal de Meleda. Nevertheless, it remains unclear whether NPPK represents an independent condition with pathogenic variations in a gene other than *SLURP1*, or whether it is a milder form of Mal de Meleda resulting from a reduction in SLURP1 expression due to pathogenic variations at the promoter region or deep intron. It was thus necessary to identify the pathogenic variations in NPPK.

## 5 IDENTIFICATION OF PATHOGENIC VARIANTS OF *SERPINB7* IN NPPK

In 2013, homozygous or compound heterozygous pathogenic variations of *SERPINB7* were identified in 13 individuals with NPPK by Akiharu Kubo and his colleagues of Keio University, where Masaji Nagashima first described the disease.^14^ The initial analysis was conducted on three individuals, exhibiting all the typical clinical manifestations of NPPK. Considering the assumption of an autosomal recessive disease, the whole exome data were subjected to analysis, which revealed the presence of 693, 677, and 747 genes in each individual that were either homozygous or compound heterozygous for variations, with a prevalence of less than 1% in the 1000 genome data set, including a considerable number of non‐pathogenic single nucleotide polymorphisms. However, only one of these genes, *SERPINB7*, which encodes a serine protease inhibitor (SNP), exhibited a variation that was common to all three cases. Furthermore, a series of variations in *SERPINB7* were identified in a series of 10 additional cases provided by Yoshihiko Mitsuhashi that were subsequently recruited for the study, confirming that the pathogenic variations of *SERPINB7* cause NPPK. Finally, the independence of the disease was confirmed, and it was registered in the Online Mendelian Inheritance in Man database in 2014 (MIM 615598).

Of note, the c.796C > T variation was identified in 19 of 26 *SERPINB7* alleles from 13 NPPK individuals in the study.^14^ The 1000 Genomes Project Phase 3 data showed that c.796C > T (rs142859678) was detected in East Asians with an allele frequency of 0.012 (12/1008 allele) and genotype frequency of 0.024 (12/504 individuals), but not in Africans (661 individuals), Europeans (503 individuals), Americans (347 individuals), or South Asians (489 individuals). East Asian individuals with the c.796C > T variant include Japanese (3/104 individuals), Han Chinese in Beijing (5/103 individuals), and Kinh in Ho Chi Minh, Vietnam (1/99 individuals). These suggest that the c.796C > T variant is a founder variant that has spread from the common ancestor to the East Asian population. This may be one of the reasons why NPPK has only been reported from Japan and not from other areas of the world. In 2014, seven cases of NPPK were reported for the first time from the country other than Japan, China, and 10 of the 14 alleles were identified as the c.796C > T variation.^15^ The c.796C > T variation was reported in 2022 from Arab‐Muslim individuals who suffered with NPPK.^16^ It is a possibility that the founder variant of East Asia may have traveled to the Arab populations because of trade activities conducted along the Silk Road.

## 6 FUNCTIONS AND DISEASES ASSOCIATED WITH SERPINs

The serpin gene is a member of the serine protease inhibitor superfamily, which is widely conserved from bacteria and archaea to vertebrates. The human genome encodes nine clades of serpin, designated A to I. SERPINB7 is a member of the clade B serpin. Most serpins that have been documented to date have protease inhibitor activity. Some have been observed to inhibit proteases other than serine proteases, including cysteine proteases.^17^, ^18^

Protease inhibitors are typically stable proteins that are highly resistant to degradation by proteases. This is not unexpected, given that they are unable to function as effective inhibitors if they are readily susceptible to protease‐mediated degradation. It is conceivable that this is the reason some serpins have lost their protease inhibitor activity during the evolutionary process and have been reprogrammed as protein storage proteins. For example, chicken albumin (ovalbumin) is a homolog of the clade B serpins, including SERPINB7. Consequently, clade B serpin is also designated “ov‐serpin.”^17^, ^18^

Pathological conditions of humans resulting from pathogenic variations in SERPIN can be classified into two primary categories. The first category is characterized by the loss of protease inhibitor activity, which leads to the uninhibited activity of the target protease and subsequent tissue damage. An illustrative example is SERPINF2 (alpha 2 antiplasmin) deficiency (MIM 262850), which manifests as a bleeding tendency due to the excessive protease activity of plasmin and increased fibrinolysis. The second category is typified by the intracellular formation of aggregates or polymers from SERPIN that have misfolded as a result of the pathogenic variation. This process leads to cellular damage and tissue damage, and these diseases are collectively referred to as “serpinopathies.” An illustrative example is familial encephalopathy (MIM 604218), in which pathogenic variations in *SERPINI1* (neuroserpin) result in the formation of neuroserpin inclusions in neurons, leading to neurodegeneration. Pathogenic variations in *SERPINA1* (α1‐antitrypsin) (MIM 613490) result in the formation of a polymer of variant SERPINA1 in the rough endoplasmic reticulum in hepatocytes that produce SERPINA1, which ultimately leads to cell damage, liver injury, and cirrhosis. In addition to cirrhosis, the amount of SERPINA1 secreted from hepatocytes into the bloodstream is reduced, leading to tissue destruction in the lungs due to excessive neutrophil elastase activity. This is caused by decreased protease inhibitor activity resulting from SERPINA1 deficiency, which in turn results in emphysema.

## 7 “WATER SIGN” AND POSSIBLE BARRIER DEFECT IN THE STRATUM CORNEUM

**FIGURE 3:**
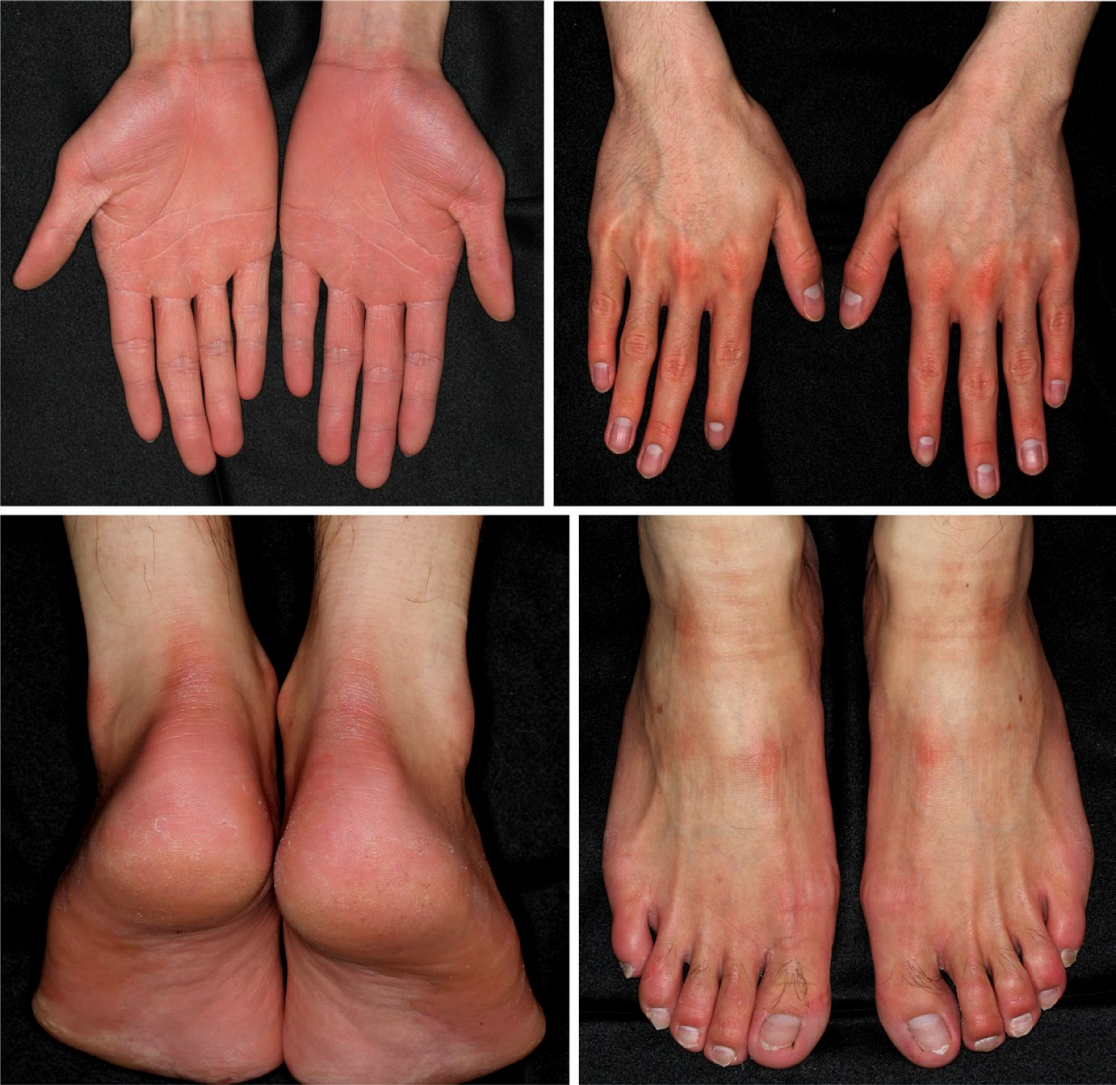
Palmoplantar redness and mild hyperkeratosis with transgrediens in Nagashima‐type palmoplantar keratosis. The hyperkeratosis is less severe, and skin redness is more pronounced than in Mal de Meleda (Figure 1). The redness extends to the dorsal surface of the toes, fingers, and the Achilles tendon areas (transgrediens), while the central dorsal area of the hands remains unaffected.

**FIGURE 4:**
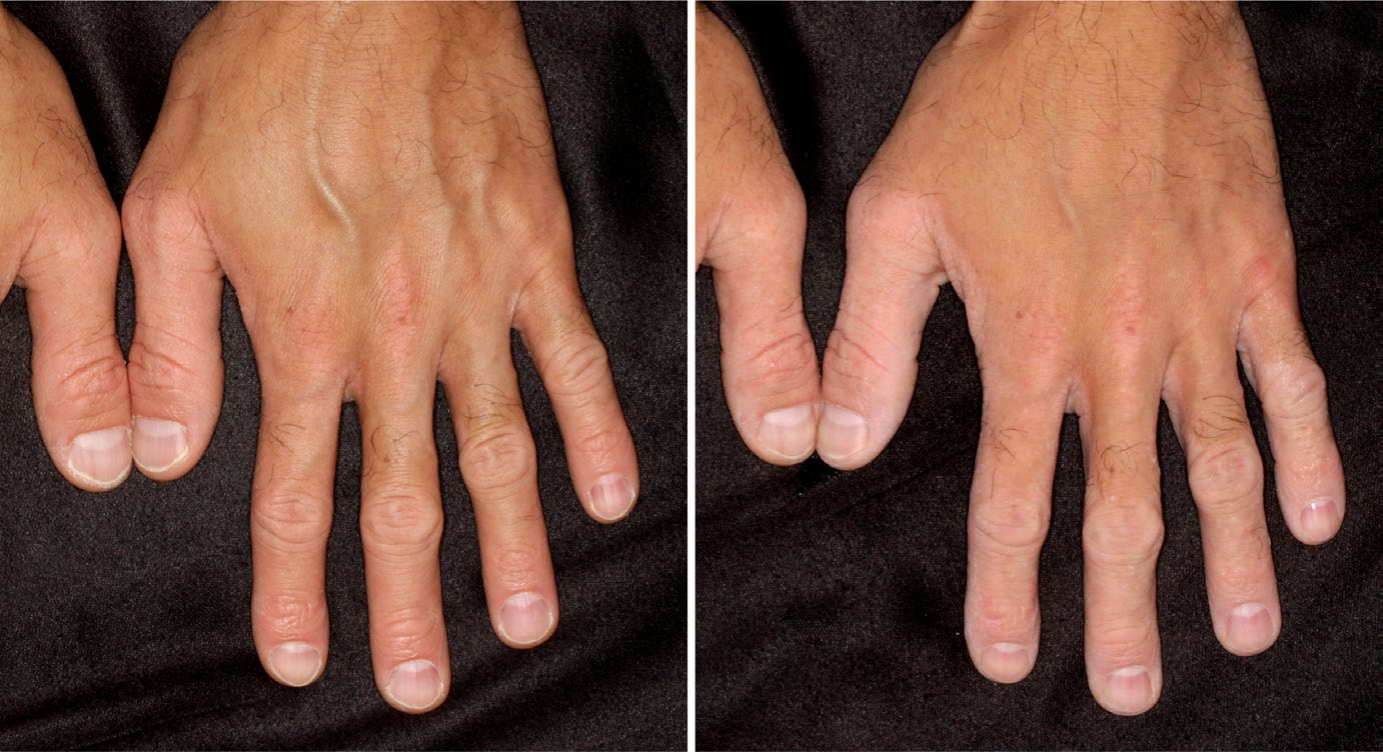
“Water sign” of Nagashima‐type palmoplantar keratosis (NPPK). Well‐demarcated whitish change that is limited in the erythematous hyperkeratotic skin lesions of NPPK.

The primary symptom of NPPK is the appearance of redness and mild hyperkeratosis on the palms and soles with transgrediens, which extends to the dorsal skin of the fingers and toes, the inner wrists, the Achilles tendon area, and the knees and elbows (Figure 3). The initial presentation of erythema and mild hyperkeratosis is typically observed by parents during the first year of the child's life. The area of erythema and hyperkeratosis gradually expands during childhood. Some patients may also present with recurrent peeling of the stratum corneum on the palms and soles (Figure 3).^16^ Following the identification of *SERPINB7* as the disease‐associated gene in 2013, Kubo et al. observed that the area of the skin exhibiting erythema and mild hyperkeratosis displayed a whitish change shortly after exposure to water and subsequently reverted to its original state upon drying in all 13 affected individuals.^14^ It is postulated that the whitish change is caused by increased water penetration into the stratum corneum due to a barrier defect induced by overactivation of target proteases. This phenomenon, which we term the “water sign,” is a diagnostic hallmark of NPPK (Figure 4) as it has been observed by the majority of parents of infant patients after exposure to water following bathing and swimming.

## 8 LOSS‐OF‐FUNCTION VARIANTS IN *SERPINA12* CAUSING PHENOCOPIED PPK WITH NPPK

In 2020, loss‐of‐function variants in *SERPINA12*, another SERPIN expressed in the epidermis, were reported to cause autosomal recessive PPK, which exhibits a phenocopy with NPPK.^19^, ^20^, ^21^ In both NPPK and SERPINA12‐related PPK, no abnormal aggregates or polymers from pathogenic SERPIN variants were observed in the patient's tissue. This indicates that the disease phenotype is not caused by the accumulation of mutant SERPINs but caused by the overactivation of the target protease, which is induced by the decreased protease inhibitory activities of SERPINB7 and SERPINA12. While clade A SERPIN is secreted from the cell and inhibits target protease in the extracellular space, clade B SERPINs are regarded as intracellular SERPIN.^17^ However, several clade B SERPINs have been demonstrated to be secreted extracellularly. For example, SERPINB1 is secreted from hepatocytes, and SERPINB2 is secreted from endothelial cells.^22^, ^23^ As the disease phenotype is quite similar between NPPK and SERPINA12‐related PPK, it is reasonable to hypothesize that these two protease inhibitors share some localization and functions that are associated with the pathogenesis.

## 9 CANDIDATE PROTEASES OVERACTIVATED IN NPPK AND SERPINA12‐RELATED PPK

A reduction in SERPINA12 expression has been demonstrated to result in a decrease in SERPINA12‐mediated inhibition of kallikrein 7 activity as well as a reduction in levels of desmoglein‐1 and corneodesmosin, two known kallikrein 7 substrates.^19^ The data indicate that SERPINA12 is secreted from keratinocytes and functions to inhibit the degradation of corneodesmosome by preventing the activity of kallikrein proteases within the extracellular space of the stratum corneum. It is, therefore, proposed that a deficiency in SERPINA12 induces stratum corneum barrier defects via overdegradation by uncontrolled kallikrein proteases. It is currently unclear whether SERPINB7 functions at the intracellular or extracellular level within the epidermis. The biopsied NPPK plantar skin exhibited acral peeling with mislocalization of desmosomal proteins.^16^ Moreover, it has been demonstrated that the silencing of SERPINB7 in normal human keratinocytes results in a reduction in the expression of desmoglein‐1 and desmocollin‐1.^16^ This suggests that a similar underlying pathogenesis may be responsible for both NPPK and SERPINA12‐related PPK. It has recently been demonstrated that legumain protease is inhibited by Serpinb7 in mice and cultured keratinocytes.^24^ Legumain is a cysteine protease that localizes primarily in endosomes and lysosomes. It would be beneficial to investigate in future studies whether overactivation of legumain induces stratum corneum barrier defects and whether a double knockout of legumain and Serpinb7 cancels the phenotype of Serpinb7 knockout mice. Furthermore, it is necessary to explore other target proteases inhibited by SERPINB7 to fully understand the pathogenesis of NPPK.

## 10 ASSOCIATION OF A FOUNDER VARIANT OF *SERPINB7* IN FINLAND WITH ATOPIC DERMATITIS

Initially, three pathogenic founder variants (c.796C > T, c.455‐1G > A, and c.218_219delAGinsTAAACTTTACCT) of *SERPINB7* (NM_003784) were identified in Japanese populations as being associated with NPPK. Subsequently, a number of founder variants of *SERPINB7* for NPPK were identified in East Asian populations.^15^, ^25^ Several reports have indicated that the complication of atopic dermatitis with NPPK is frequently observed.^26^, ^27^ However, it remains unclear whether the observed association between NPPK and atopic dermatitis is due to the high prevalence of both diseases in East Asian populations or to a direct causal relationship. In 2020, the initial founder variant of NPPK in Western populations, *SERPINB7* (NM_003784.3) c.1136G > A (p.Cys379Tyr, rs201208667), was identified in Finland.^28^ Subsequently, a GWAS study using a cohort from Finland, Estonia, and the UK identified an SNP (rs188720898) located near *SERPINB7* as a modulator of susceptibility for atopic dermatitis and found the association of the SNP with the Finnish NPPK founder variant in high linkage disequilibrium.^29^ It is reasonable to hypothesize that the compromised stratum corneum barrier resulting from the overactivation of target proteases accelerates percutaneous sensitization and allergic inflammation by enhancing the penetration of allergens, activating innate lymphoid cells, and/or inducing dysbiosis of skin commensal bacteria due to structural and immunological alterations of the stratum corneum. The underlying mechanisms of the association of atopic dermatitis with NPPK will be elucidated in future studies.

## 11 CURRENT UNDERSTANDING OF SYMPTOMS AND TREATMENT OF NPPK

In both NPPK and SERPINA12‐related PPK, erythematous hyperkeratosis is the most prominent symptom, exhibiting transgrediens characteristics. The expression of both SERPINB7 and SERPINA12 has been observed in the epidermis of the skin on the entire body surface. However, the skin symptoms appear in a limited area, and the underlying mechanisms of this phenomenon remain unclear. Furthermore, the distinguishing characteristics between affected and unaffected skin have yet to be fully elucidated.

Itchiness and eczema are not a direct consequence of NPPK; rather, they are precipitated by complications with atopic dermatitis or tinea pedis. It is, therefore, imperative that appropriate therapeutic intervention be instituted in cases where NPPK patients present with pruritus or exacerbation of cutaneous symptoms, which are predominantly attributable to eczema or tinea pedis. It is noteworthy that NPPK patients frequently experience recurrent tinea pedis, which bears resemblance to Mal de Meleda. Therefore, if the patient's symptoms are exacerbated or if pruritus emerges, it may be caused by a complication of tinea pedis and should be treated accordingly. It is unfortunate that the redness of the skin remains untreatable and often reduces the patients' quality of life (QOL). The use of cosmetics such as Covermark® is an effective method of covering up the redness, especially in adolescents. Another problem that reduces patients' QOL is the odor of the soles and sometimes of the palms. Extensive washing and the use of deodorants are effective in reducing the odor.

For genetic therapy, gentamicin‐induced readthrough has been attempted to skip the East Asian founder mutation, c.796C > T (p.Arg266Ter), to produce functional SERPINB7.^30^

## 12 CONCLUSION AND FUTURE PERSPECTIVE

A decade has elapsed since the identification of pathogenic variants of *SERPINB7* as the underlying cause of NPPK. However, the molecular mechanisms that give rise to the major clinical symptoms, including skin redness, hyperkeratosis, odor, and fungal susceptibility, remain poorly understood. It is hoped that the identification of all of the proteases inhibited by SERPINB7 and their substrates as well as the structural and immunological changes that occur in the lesional skin, will elucidate the molecular pathogenesis and lead to the development of disease‐specific treatments. Furthermore, it is hoped that molecular therapies will be developed that directly modify the genetic alterations.

## CONFLICT OF INTEREST STATEMENT

Akiharu Kubo is an Editorial Board member of *The Journal of Dermatology* and an author of this article. To minimize bias, he was excluded from all editorial decision‐making related to the acceptance of this article for publication.

## ETHICS STATEMENT

This study was approved by the Ethics Committee of Kobe University (B230011), Keio University School of Medicine (20 120 226 and 20 236 014), and National Center for Child Health and Development (926, 2020–326 and 2023–027), in accordance with the Declaration of Helsinki.

## INFORMED CONSENT

Written informed consent was obtained from all individuals for the publication of the case details and clinical images.
